# Supplementary material for: Complexome profiling on the Chlamydomonas lpa2 mutant reveals insights into PSII biogenesis and new PSII associated proteins
Source: J Exp Bot. 2021 Aug 26;73(1):245–62. doi: 10.1093/jxb/erab390 (PMC8730698; doi:10.1093/jxb/erab390)
Supplement: erab390_suppl_Supplementary_Dataset_S1 [file erab390_suppl_supplementary_dataset_s1.zip › Supplemental Dataset 1 - Excel List and all profiles/plots/FNR1_Cre11.g476750.html]

Trivial name: FNR1  
  
Euclidean distance: 190784.69  
Mean Intensity (WT): 24189.79  
Mean Intensity (Mut): 31146.40  
Distance: 6.13  
  
MapMan: OPP.electron transfer;PS.lightreaction.other electron carrier (ox/red).ferredoxin reductase  
  
p value of intensity sums Welch test: 0.1750
